# Perioperative multivessel coronary artery spasm and cardiac arrest after cardiac surgery

**DOI:** 10.1093/icvts/ivae085

**Published:** 2024-05-02

**Authors:** Chen Chen, Yi Chen, Xiao Zhang, Liang Zou

**Affiliations:** Department of Cardiovascular Surgery, Fuwai Hospital, National Center for Cardiovascular Diseases, Chinese Academy of Medical Sciences and Peking Union Medical College, Beijing, China; National Clinical Research Center for Cardiovascular Diseases, State Key Laboratory of Cardiovascular Disease, Fuwai Hospital, National Center for Cardiovascular Diseases Chinese Academy of Medical Sciences and Peking Union Medical College, Beijing, China; Department of Cardiovascular Surgery, Fuwai Hospital, National Center for Cardiovascular Diseases, Chinese Academy of Medical Sciences and Peking Union Medical College, Beijing, China; National Clinical Research Center for Cardiovascular Diseases, State Key Laboratory of Cardiovascular Disease, Fuwai Hospital, National Center for Cardiovascular Diseases Chinese Academy of Medical Sciences and Peking Union Medical College, Beijing, China; Department of Cardiovascular Surgery, Fuwai Hospital, National Center for Cardiovascular Diseases, Chinese Academy of Medical Sciences and Peking Union Medical College, Beijing, China; National Clinical Research Center for Cardiovascular Diseases, State Key Laboratory of Cardiovascular Disease, Fuwai Hospital, National Center for Cardiovascular Diseases Chinese Academy of Medical Sciences and Peking Union Medical College, Beijing, China; Department of Cardiovascular Surgery, Fuwai Hospital, National Center for Cardiovascular Diseases, Chinese Academy of Medical Sciences and Peking Union Medical College, Beijing, China; National Clinical Research Center for Cardiovascular Diseases, State Key Laboratory of Cardiovascular Disease, Fuwai Hospital, National Center for Cardiovascular Diseases Chinese Academy of Medical Sciences and Peking Union Medical College, Beijing, China

**Keywords:** Coronary artery spasm, Cardiac surgery, Perioperative complications, Cardiac arrest

## Abstract

Postoperative coronary artery spasm, a rare but potentially fatal complication following cardiac surgery, warrants significant attention. This report discusses a 64-year-old male who suffered a severe coronary artery spasm leading to cardiac arrest following surgery. Initially stable, the patient rapidly developed critical ventricular arrhythmias and hypotension, resulting in cardiac arrest 4 h post-surgery. Emergency coronary angiography revealed extensive spasms, successfully managed with intracoronary nitroglycerine. This case stresses prompt recognition and management of coronary artery spasm after non-coronary cardiac procedures, underscoring coronary angiography's vital role in diagnosis and treatment.

## INTRODUCTION

Coronary vasospasm following cardiac surgery, a relatively rare yet critical condition, can precipitate myocardial infarction and circulatory collapse if not promptly managed [1]. The incidence of refractory vascular spasm following coronary artery bypass grafting procedures ranges from 0.8% to 1.3% [2]. Nonetheless, there is a paucity of literature on coronary artery spasm following other cardiac surgeries. We present a severe case of multivessel coronary artery spasm occurring after the repair of a partial anomalous pulmonary venous connection (PAPVC), atrial septal defect repair (ASD) and tricuspid valve annuloplasty, which led to cardiac arrest. This case offers novel insights into perioperative coronary vasospasm, emphasizing its severity and the imperative for heightened clinician awareness.

## CASE REPORT

**Figure 1:**
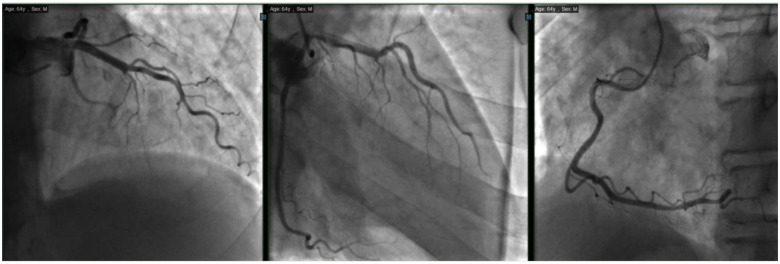
Preoperative coronary angiogram images revealing normal coronary arteries.

A 64-year-old male presented with progressive exertional dyspnoea. Diagnostic investigations identified a PAPVC and a 17-mm ASD. Preoperative angiography displayed normal coronary arteries (Fig. 1). Transthoracic echocardiography indicated moderate tricuspid regurgitation with a left ventricular ejection fraction of 70%. Consequently, the patient was scheduled for surgical repair of the PAPVC, ASD and tricuspid valve annuloplasty.

**Figure 2:**
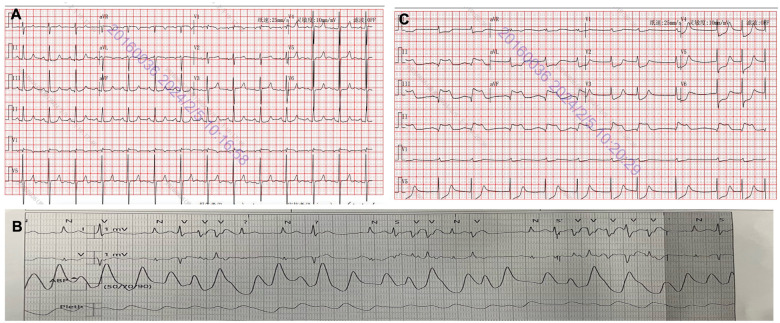
Dynamic variants of ECG. (**A**) Initial 12-lead ECG at ICU admission showed normal sinus rhythm. (**B**) Telemetry data 4 h postoperatively revealed ventricular arrhythmias. (**C**) Following ECMO intervention, ECG exhibited significant ST-segment depression in inferior limb leads with reciprocal ST-segment elevations in anterior chest leads. ECG: electrocardiography; ICU: intensive care unit; ECMO: extracorporeal membrane oxygenation.

The surgery was successful. The postoperative intensive care unit (ICU) regimen included intravenous potassium and magnesium supplementation, continuous low dose (<2 μg/kg/min) dopamine infusion, ensuring stable haemodynamics in the patient. Initial postoperative indices showed a high-sensitivity cardiac troponin I level of 0.766 ng/ml, blood gas analysis revealed potassium ion at 4.95 mmol/l and magnesium ion at 0.8 mmol/l, with an electrocardiography (ECG) (Fig. 2A) and echocardiogram within normal limits. However, 4 h postoperatively, the patient experienced frequent ventricular arrhythmias (Fig. 2B) and hypotension, culminating in a sudden cardiac arrest. Resuscitation attempts employing an extracorporeal membrane temporary pacemaker proved ineffective. Despite the implementation of external chest compressions and intermittent administration of epinephrine, these measures failed to re-establish cardiac rhythm and blood pressure. An emergent bedside thoracotomy was conducted, revealing no structural cardiac abnormalities. Persistent ventricular tachycardia and fibrillation were observed under extracorporeal circulation, necessitating the deployment of extracorporeal membrane oxygenation (ECMO) and an intra-aortic balloon pump to facilitate weaning from the extracorporeal circuit. Subsequent ECG (Fig. 2C) showed atrial fibrillation with ST-segment changes and abnormal Q waves. Echocardiography indicated a significant reduction in left ventricular ejection fraction to 20% and a marked increase in high-sensitivity cardiac troponin I levels to 44.8 ng/ml.

Given the suspicion of coronary artery occlusion, a coronary angiography was performed 13 h post-surgery. The angiography uncovered diffuse multivessel coronary artery spasms without thrombus or dissection (Video 1). Post-intracoronary nitroglycerine administration resolved the spasms, revealing no residual stenosis (Video 2). Post-angiography, the patient encountered acute kidney injury, necessitating continuous renal replacement therapy. Despite ECMO support, the patient developed coagulopathy and persistent circulatory failure, culminating in mortality the following day.

## DISCUSSION

Postoperative coronary vasospasm, rare but perilous, poses significant risks of haemodynamic instability and fatal ventricular arrhythmias. Its prevalence remains unclear. To our current understanding, this case represents the first documented instance of multivessel coronary artery spasm leading to sudden cardiac arrest shortly after surgical correction of PAPVC, ASD and tricuspid valve annuloplasty.

The etiopathogenesis of perioperative coronary vasospasm is linked to several factors. In cases with preoperative angiography showing normal coronary arteries, a combination of increased oxidative stress, inflammation, platelet-mediated vasoconstrictive agent release and changes in vascular reactivity are thought to contribute to the development of postoperative coronary artery spasm [3, 4]. During the early postoperative phase following cardiac surgery, potential myocardial depression may necessitate the use of vasoactive agents like dopamine to maintain adequate cardiac output and blood pressure. However, it is essential to recognize that the administration of these agents carries inherent risks of precipitating arrhythmias and coronary artery spasms. Literature [5] emphasizes the importance of utilizing vasoactive agents with a lower likelihood of causing arrhythmias and vasospasm, such as vasopressin and norepinephrine.

In the context of acute postoperative circulatory instability, prompt recognition and targeted intervention for coronary vasospasm are vital. In this case, emergency coronary angiography, prompted by ventricular arrhythmias and cardiac arrest, unveiled diffuse coronary artery spasm. Despite its rarity, if not swiftly addressed, coronary vasospasm following cardiac surgery can be fatal. The use of the intra-aortic balloon pump is crucial for enhancing coronary flow in severe cases, and the employment of ECMO provides critical support in instances of severe cardiogenic shock or cardiac arrest, effectively addressing left ventricular dysfunction and associated metabolic disturbances [2].

In conclusion, this case highlights the necessity of considering postoperative coronary spasm as a potential cause of severe cardiogenic shock or cardiac arrest. It emphasizes the relevance of postoperative coronary spasm not just in coronary bypass surgery but also following surgeries for congenital heart diseases. Additionally, it underscores the crucial role of coronary angiography in diagnosing and managing this condition.

### Limitations

Utilizing ultrasound assessment for evaluating pulmonary arterial hypertension is limited compared to the gold standard of cardiac catheterization.

## Data Availability

All relevant data are within the manuscript and its Supporting Information files.
